# Therapeutic potential of common Phytoestrogens found in traditional Chinese medicine in chronic kidney diseases

**DOI:** 10.3389/fphar.2025.1599097

**Published:** 2025-11-19

**Authors:** Tongtong Liu, Minghan Li

**Affiliations:** 1 Heilongjiang University of Chinese Medicine, Harbin, China; 2 Dalian Medical University, Dalian, China

**Keywords:** chronic kidney disease, phytoestrogens, traditional Chinese medicine, treatment, research progress

## Abstract

In recent years, phytoestrogens in traditional Chinese medicine (TCM)—a class of estrolike active components naturally occurring in medicinal plants—have gradually emerged as a research hotspot in the treatment of various diseases due to their multi-target regulatory potential. These metabolites are abundant in TCM, and an increasing body of evidence indicates that they have beneficial effects in chronic kidney diseases. Research demonstrates that phytoestrogens can alleviate renal pathological damage by regulating the expression of pro-inflammatory cytokines, reducing oxidative stress, and inhibiting the activation of fibrosis pathways. The application of phytoestrogens as a therapeutic strategy for chronic kidney diseases is highly promising. This review comprehensively summarizes the status of TCM phytoestrogens in chronic kidney disease research and elaborates in detail on various types of these compounds, such as baicalin and puerarin, as well as their protective effects on chronic kidney disease observed in animal and cell experiments. Additionally, we highlight the advantages of TCM phytoestrogens in the regulation of chronic kidney disease and discuss their potential clinical significance and future research directions in this field. These findings will provide a promising avenue for the development of drugs aimed at treating chronic kidney disease.

## 1 Introduction

Chronic kidney disease (CKD) has emerged as a significant public health challenge globally. Approximately 850 million people worldwide are affected by CKD. The incidence rate varies significantly across different regions and countries and has been trending upward each year (Kovesdy, 2022; Qin et al., 2024). The characteristics of CKD include a progressive decline in renal function, which significantly increases the risk of all-cause mortality (Xie et al., 2018). Epidemiological studies indicate that there are gender differences in chronic kidney disease CKD (Ricardo et al., 2019). Women have a lower risk of CKD progression and mortality compared to men, who are more likely to progress to end-stage renal disease (ESRD) (Inada et al., 2016; Smith et al., 2025). This disparity may be associated with sex hormones, particularly estrogen (Valdivielso et al., 2019).

Estrogen is a class of steroid hormones that play crucial physiological roles. It is primarily produced by the ovaries and adrenal glands (Lee et al., 2012). In addition to its crucial role in the female reproductive system, estrogen is involved in the regulation of various systems, including whole-body glucose and lipid metabolism, bone health, the nervous system, the cardiovascular system, the renal endocrine system, the digestive system, and the immune system (Clemenza et al., 2022; Critchlow et al., 2023; Wilkinson and Hardman, 2021). Studying populations with abnormal endogenous estrogen status, such as patients with gonadal dysfunction, can yield deeper insights into the protective role of estrogen. Mayer-Rokitansky-Kuster-Hauser (MRKH) syndrome and 46, XX gonadal dysgenesis are two such conditions that are often managed with estrogen supplementation. In addition to supporting the reproductive system in maintaining pubertal development and establishing a normal menstrual cycle, estrogen exerts protective effects on the skeletal and cardiovascular systems. These effects include preventing fractures, lowering blood pressure, regulating lipid distribution, and reducing the risk of thromboembolism (Kapczuk et al., 2016; Yavas Abalı and Guran, 2024). In recent years, research has demonstrated that estrogen can protect the kidneys through multiple mechanisms, including the regulation of extracellular matrix metabolism, the renin-angiotensin system (RAS), nitric oxide levels, antioxidant effects, inhibition of inflammatory responses, and promotion of the expression of matrix metalloproteinases (Guccione et al., 2002; Tanaka et al., 2013; Wu et al., 2016). However, the application of estrogen also presents several side effects, such as stimulation of the gastrointestinal tract, an increased risk of thrombosis, and the potential risks of breast cancer, endometrial cancer, and venous thrombosis (An, 2016). Therefore, the importance of understanding the renal protective mechanisms of estrogen and developing new therapeutic strategies is self-evident. Traditional Chinese Medicine (TCM) has unique concepts in the prevention and treatment of chronic kidney disease. Previous studies have shown that traditional Chinese botanical drug is rich in phytoestrogens, which can effectively inhibit renal inflammation and fibrosis processes to protect the kidneys, alleviate kidney damage, and improve renal function (Zhao et al., 2025). Phytoestrogens are a group of metabolites with estrogen-like effects, whose structures are like endogenous steroid estrogens. In particular, the hydroxyl group on the phenolic ring corresponds to the hydroxyl group on the aromatic ring of estrogens, allowing them to bind to estrogen receptors and exert their effects (Ceccarelli et al., 2022). Recent studies have indicated that phytoestrogens not only activate the classical estrogen receptors ERα and ERβ (Chen X. et al., 2016; Pepermans et al., 2021), but also specifically bind to G protein-coupled estrogen receptors (GPER) (Carmeci et al., 1997; McLaughlin and De Vries, 2001; Rae and Johnson, 2005; Thomas and Dong, 2006; Thomas et al., 2005). Furthermore, phytoestrogens can also exert effects through non-estrogen receptor-mediated mechanisms, such as activating the phosphatidylinositol 3-kinase/protein kinase B (PI3K/Akt) signaling pathway to reduce apoptosis, regulating the expression of nuclear factor Kappa B (NF-κB) and mitogen-activated protein kinase (MAPK) to alleviate inflammatory responses, and activating antioxidant protein gene expression to exert antioxidant effects (Goh et al., 2022; Gorzkiewicz et al., 2021; Kim, 2021).

Based on the latest developments, this article aims to thoroughly investigate the mechanisms of action of estrogen and its receptors in CKD, analyze their structure, function, and regulatory mechanisms, and emphasize the role of phytoestrogens from TCM in the context of CKD. Through comparative studies of estrogen and phytoestrogens, this research will provide additional avenues for potential therapeutic interventions in CKD.

## 2 Literature search and methods

The primary objective of this study is to summarize and analyze the protective effects and molecular mechanisms of phytoestrogens in CKD. We will focus on evidence from *in vitro*, *in vivo*, and existing clinical studies; this will help elucidate how phytoestrogens modulate kidney injury and associated signaling pathways. A comprehensive literature search was conducted across the PubMed and Web of Science databases. Search terms included “phytoestrogen,” “flavonoid,” “coumarin,” “lignan,” “stilbene,” “terpenoid,” “sterol,” and related metabolites such as baicalin, puerarin, resveratrol, schisandrin A/B, ginsenoside Rb1/Rh1, and dioscin. These were combined with “chronic kidney disease,” “renal fibrosis,” and “diabetic nephropathy”. The search was conducted up to January 2025.

The inclusion criteria prioritize original research articles (*in vitro*, *in vivo*, and clinical studies) and high-quality reviews published in English that examine the effects and mechanisms of phytoestrogens on renal injury and fibrosis. Exclusion criteria include unpublished works, abstracts, and studies not directly relevant to the core focus of this study.

Additionally, we considered the non-specific reactivity of PAINS (pan assay interfering compounds), which can lead to false-positive results in in vitro experiments. To assist readers in identifying and interpreting experimental results that may be affected by interference, we have flagged all metabolites mentioned in the text with PAINS risk in Table 1. All plant-derived species mentioned in the text have been taxonomically validated using the MPNS portal (http://mpns.kew.org/mpns-portal/), with their complete scientific names (including authoritative nomenclature and taxonomic classification) provided.

**TABLE 1:** Phytochemical-containing traditional Chinese medicines as partial agonists of estrogen receptors.

Classification	Phytochemical	CAS	Structure	Source	Pains
Flavonoid	Baicalin	21967-41-9	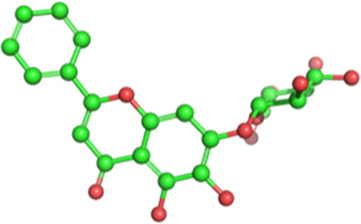	*Scutellaria baicalensis* Georgi (Lamiaceae)	Yes
	Puerarin	3681-99-0	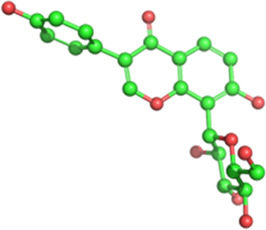	*Pueraria montana* var. *lobata* (Willd.) Sanjappa & Pradeep (Fabaceae)	No
Coumarin	Angelicin	523-50-2	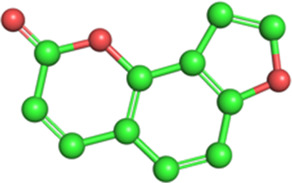	*Angelica archangelica* L. (Apiaceae)	No
	Psoralen	66-97-7	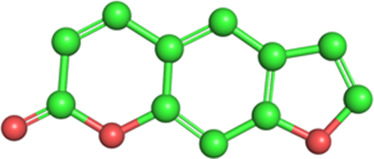	*Cullen corylifolium* (L.) Medik. (Fabaceae)	No
	Osthole	484-12-8	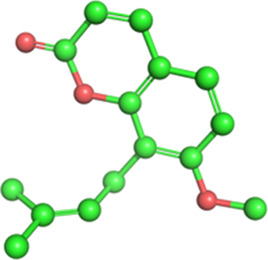	*Cnidium monnieri* (L.) Cusson (Apiaceae)	No
Lignans	Schisandrin A	61281-38-7	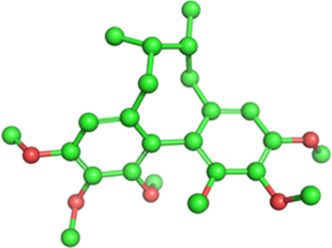	*Schisandra chinensis* (Turcz.) Baill. (Schisandraceae)	No
	Schisandrin B	61281-37-6	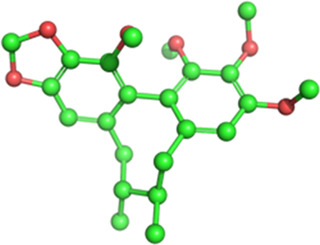		No
Stilbene	Resveratrol	501-36-0	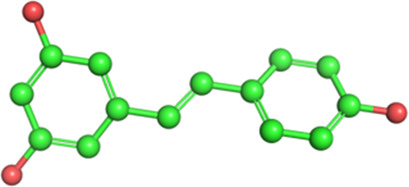	*Reynoutria japonica* Houtt. (Polygonaceae)	No
	2,3,5,4′-Tetrahydroxystilbene-2-O-β-D-glucoside	82373-94-2	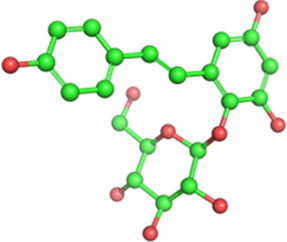	*Reynoutria multiflora* (Thunb.) Moldenke (Polygonaceae)	No
	Physcion	521-61-9	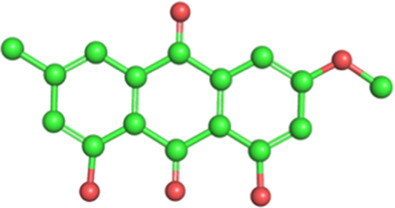		Yes
Terpenoid	Ginsenoside Rb1	41753-43-9	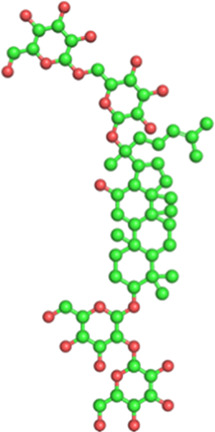	*Panax ginseng* C.A.Mey. (Araliaceae)	No
	Ginsenoside Rh1	63223-86-9	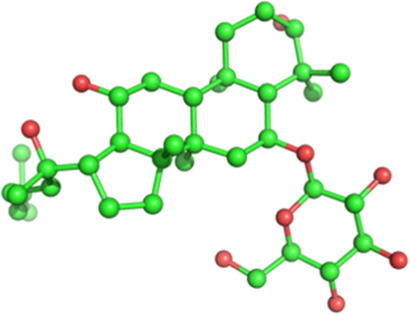		No
Sterol	Dioscin	19057-60-4	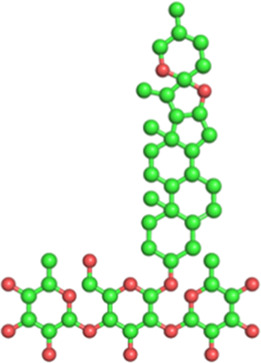	*Dioscorea panthaica* Prain & Burkill (Dioscoreaceae)	No

The structures of phytochemicals were developed using PyMOL.

## 3 Pathophysiology of chronic kidney disease

Chronic kidney disease is a complex clinical syndrome whose pathophysiological mechanisms involve multiple factors. Kidney injury of pre-renal, renal, and post-renal types can all lead to CKD (Kalantar-Zadeh et al., 2021; Nørregaard et al., 2023; Zhao et al., 2021). Pre-renal causes mainly involve hemodynamic changes, such as low blood volume, reduced cardiac output, or systemic hypotension, which result in inadequate renal perfusion and consequently reduce the glomerular filtration rate (GFR) (Master Sankar Raj et al., 2015; Polichnowski, 2018). Studies have indicated that pre-renal factors are particularly common among CKD patients, especially in those with cardiovascular disease or diabetes, where pre-renal hypoperfusion may accelerate renal function deterioration (Dilsizian et al., 2021; Malleshappa and Shah, 2015). Moreover, pre-renal causes can also lead to acute kidney injury (AKI), and AKI and CKD are closely interacted. AKI not only increases the risk of CKD but may also accelerate its progression (Guan et al., 2022; Li et al., 2022). Renal causes directly involve pathological changes in the kidneys, including glomerular diseases, tubulointerstitial lesions, and vascular diseases. Diabetic nephropathy and hypertensive nephropathy are major etiologies of CKD, characterized by pathological features such as glomerulosclerosis and tubulointerstitial fibrosis (Hao et al., 2024; Jia et al., 2025). Tubulointerstitial lesions are often caused by chronic inflammation, drug toxicity, or metabolic abnormalities, which further worsen renal failure by impairing tubular structure and function (Bhandari et al., 2025; Lu et al., 2023). Additionally, renal causes may also result in microvascular rarefaction and cell cycle control dysregulation, mechanisms that significantly contribute to CKD progression (Krishnan et al., 2021). Post-renal causes of chronic kidney disease (CKD) mainly involve urinary tract obstruction, such as renal stones, prostatic enlargement, or tumor compression (Saad et al., 2024). Obstruction of the urinary tract leads to increased pressure within the renal pelvis, which in turn affects the function of renal tubules and glomeruli (Li et al., 2021b; Nørregaard et al., 2023). Long-standing obstruction can cause not only renal parenchyma atrophy but also accelerate CKD progression *via* inflammatory and fibrotic mechanisms (Khater et al., 2025). It should be emphasized that in the early stage, post-renal obstruction-induced pathological changes are partially reversible upon obstruction relief. However, the pathological effects will become irreversible if timely intervention is not provided (Khater et al., 2025). The interactions among pre-renal, renal, and post-renal causes in CKD pathology are intricate. For example, pre-renal hypoperfusion may worsen renal lesions, while post-renal obstruction may further damage tubular function by increasing intrarenal pressure (Nørregaard et al., 2023). Additionally, these causes may accelerate CKD progression through common pathological mechanisms like oxidative stress, inflammation, and fibrosis.

Oxidative stress plays a central role in the progression of CKD. The excessive production of reactive oxygen species (ROS) leads to oxidative damage of lipids, proteins, and DNA, thereby activating various pro-inflammatory and pro-fibrotic signaling pathways (Liu et al., 2025). Studies have shown that oxidative stress not only directly damages renal cells but also promotes the release of inflammatory mediators, such as tumor necrosis factor-alpha (TNF-α) and interleukin-6 (IL-6), by activating transcription factors like NF-κB, thus exacerbating the inflammatory response (Rapa et al., 2019). Furthermore, oxidative stress accelerates the occurrence of cardiovascular complications, which are particularly common in CKD patients, by inducing endothelial dysfunction and vascular calcification (Baaten et al., 2023).

Similarly, the inflammatory response is another key mechanism in the progression of CKD. The chronic inflammatory state not only directly damages renal tissue but also leads to fibrosis through the activation of fibroblasts and the promotion of extracellular matrix (ECM) deposition (Panizo et al., 2021). Research indicates that pro-inflammatory factors commonly found in CKD patients, such as C-reactive protein (CRP) and IL-6, are closely related to the decline of renal function and the occurrence of cardiovascular events (Stopic et al., 2022). Additionally, inflammation further exacerbates the fibrotic process by inducing the transition of renal tubular epithelial cells to myofibroblasts through epithelial-mesenchymal transition (EMT) (Geng et al., 2025).

Fibrosis is the ultimate common pathway in the progression of CKD. Regardless of the cause-prerenal, renal, or postrenal-the outcome is renal fibrosis. This process is characterized by fibroblast activation, excessive ECM deposition, and renal structure disruption (Huang et al., 2023). Studies have established that transforming growth factor-beta (TGF-β) is a key fibrosis regulator. Through the Smad signaling pathway, TGF-β drives ECM synthesis while inhibiting its degradation, thereby promoting irreversible fibrosis progression (Li et al., 2024b). Furthermore, fibrosis also results in the destruction of the microvascular structure of the kidneys, causing loss of nephron function and further accelerating the progression of CKD (Biglari et al., 2025). In summary, prerenal, renal, and postrenal factors drive CKD progression through shared pathological mechanisms like oxidative stress, inflammation, and fibrosis.

## 4 Estrogen’s impact on chronic kidney disease

Estrogen plays a crucial role in kidney health and is associated with the progression of CKD. The relationship between estrogen levels and renal function is complex, varying across different populations and physiological conditions. Multiple studies have confirmed the protective effect of estrogen on kidney health. A population-based cohort study revealed that women who underwent bilateral oophorectomy before menopause had a higher risk of CKD, as assessed by the evaluation of glomerular filtration rate (eGFR). For younger patients, estrogen replacement therapy may provide relief (Kattah et al., 2018). Postmenopausal women experience a decrease in estrogen levels accompanied by an increase in follicle-stimulating hormone (FSH) levels. FSH enhances the expression of collagen IV, fibronectin (FN), and plasminogen activator inhibitor-1(PAI-1), stimulates the secretion of Interleukin-8(IL-8) by human kidney 2 (HK-2) cells, promotes macrophage migration, exacerbates tubulointerstitial fibrosis, and worsens kidney damage (Zhang et al., 2019). Among non-reproductive organs, the kidney exhibits one of the highest levels of estrogen receptor (ER) expression, particularly ERα (Buléon et al., 2020). The staining of human renal biopsy indicates that ERα is predominantly expressed in the glomeruli and renal tubules, while both ERα and ERβ are present in the renal proximal tubules. Several studies conducted on rodents and humans have shown that GPER1 is widely expressed in the reproductive system, cardiovascular system, renal system, brain, adrenal gland, adipocytes, and bones (Eissa and Gohar, 2023). In the renal ischemia-reperfusion model, downregulation of ERα receptors in rat kidneys leads to transforming growth factor-β (beta) receptor I (TGF-βRI) dysregulation and sma- and mad-related protein 2/3 (SMAD2/3) activation. This process triggers the production and release of downstream inflammatory factors, further exacerbating kidney damage. Conversely, estradiol can activate ERα receptors, thereby reducing renal fibrosis and inflammation (Ren et al., 2022). A reduction in ERα in proximal tubular epithelial cells exacerbates significant albuminuria, leading to tubular injury and lipid accumulation (Muroya et al., 2012). In the unilateral ureteral obstruction (UUO) and 5/6 nephrectomy mouse models, ERβ expression is significantly decreased. ERβ exerts renoprotective effects in CKD by blocking Smad3 (Cao et al., 2023).

The research conducted by Michael P. Hutchens and colleagues demonstrates that estrogen can decrease glomerular endothelial permeability following ischemia-reperfusion injury, thereby protecting renal function *via* G protein-coupled receptor 30 (Hutchens et al., 2012). This indicates that estrogen can influence disease development through its interaction with estrogen receptors. Additionally, estrogen may also mediate CKD through non-estrogen receptor pathways. For instance, in a chronic nitric oxide inhibition model that exacerbates CKD progression, male rats exhibit significantly greater albuminuria, histological damage, interstitial inflammation, and tubular interstitial fibrosis compared to female rats. This phenomenon is attributed to the lower estrogen levels in male mice, which leads to the hyperactivation of the renin-angiotensin-aldosterone system (Fanelli et al., 2017). However, research results regarding the effects of exogenous estrogen are mixed. While some studies indicate beneficial outcomes, long-term hormone replacement therapy carries potential risks. For instance, elevated proteinuria and a reduced glomerular filtration rate may lead to renal injury (Zimmerman et al., 2017). Additionally, estrogen has been found to influence other hormonal factors that are crucial in the progression of kidney diseases. For instance, it regulates renin synthesis and affects homocysteine metabolism, with homocysteine serving as a marker associated with cardiovascular risk in CKD (Niu et al., 2022). Considering these factors, the timing of estrogen therapy is crucial for maximizing its benefits while minimizing associated risks. Current research advocates for further investigation into the mechanisms underlying estrogen’s protective effects and their implications for renal health in both sexes. The use of estrogen supplements, particularly in postmenopausal women or those who have undergone surgical menopause, must be carefully evaluated by healthcare providers when formulating treatment plans for chronic kidney disease.

## 5 The role and mechanisms of phytoestrogens

### 5.1 The bidirectional regulatory effects and limitations of phytoestrogen

In recent years, phytoestrogens, which are natural analogues of estrogen, have garnered extensive attention. These metabolites, extracted from plants, exhibit estrogen-like effects due to their structural similarity to endogenous steroid estrogens, enabling them to bind to estrogen receptors (Hsieh et al., 2018; Lóránd et al., 2010). Phytoestrogens exhibit bidirectional regulatory characteristics, can function as either estrogen agonists or antagonists, with their specific action depending on concentration and bioavailability (Rettberg et al., 2014). They can bind to estrogen receptors and exert weak estrogenic effects to compensate for the deficiency of estrogen. Alternatively, they can competitively bind to estrogen receptors with endogenous estrogen, exerting anti-estrogenic effects that inhibit the side effects of estrogen (Shelly et al., 2008). Phytoestrogens can influence the structural and functional integrity of various systems, presenting both positive and negative effects (Rietjens et al., 2017; Sirotkin and Harrath, 2014). Their beneficial effects include potential protective roles in the cardiovascular, skeletal, nervous, reproductive, and digestive systems, as well as in skin and breast health. They also show some effectiveness in pain relief (Ceccarelli et al., 2022). However, like estrogen, they may also increase the potential cancer risk in estrogen-sensitive tissues (Domínguez-López et al., 2020). Notably, phytoestrogens possess endocrine-disrupting properties, which may interfere with the hypothalamic-pituitary-thyroid axis and affect thyroid function, including the synthesis and secretion of thyroid hormones (Domínguez-López et al., 2020). However, concerns about adverse reactions mainly stem from data derived from *in vitro*, animal, or epidemiological studies, whereas clinical investigations generally report no significant adverse events (Rietjens et al., 2017). Nonetheless, when applying these findings, we focus on reviewing the known thyroid impacts and reproductive effects.

### 5.2 Phytoestrogens and estrogen receptors: binding interactions and conformational changes

Although the chemical structure of phytoestrogens differs from that of endogenous estrogens, they can still bind to endogenous estrogen receptors and activate the associated signaling pathways to exert their various biological effects (Wang et al., 2008). ERs are classified into two intracellular subtypes: ERα and ERβ (Hilakivi-Clarke and de Assis, 2006). These receptors function similarly to nuclear transcription factors, regulating gene expression to elicit biological responses. Different phytoestrogens exhibit varying affinities for ERα and ERβ. For instance, isoflavones show approximately five times higher affinity for ERβ than for ERα (Vitale et al., 2013). Recent studies have also investigated artificially synthesized phytoestrogens, such as 4-(E)-{(4-hydroxyphenylimino)-methylbenzene,1,2-diol} (HPIMBD). HPIMBD enhances selectivity by using the stereochemical structure of its ortho-dihydroxy groups to precisely complement the ERβ binding cavity. This promotes the formation of additional hydrogen bonds, reduces binding energy, and selectively activates ERβ signaling. Unlike the first-generation phytoestrogen resveratrol, which forms only three hydrogen bonds and fails to bind effectively through its 3,5-hydroxy groups, HPIMBD offers greater stability and overcomes these limitations (Ronghe et al., 2014).

In addition to the genomic effects mediated by the intracellular α and β subtypes of estrogen receptors (ER), estradiol can also trigger rapid non-genomic signaling through the G protein-coupled estrogen receptor (GPER) (Levin, 2015; Prossnitz et al., 2008). Initially classified as an orphan receptor (Owman et al., 1996), GPER has been studied using computational simulations and molecular docking to analyze phytoestrogen binding modes. These studies show that phytoestrogens bind to GPER similarly to E2 (Ariyani et al., 2020; Luo and Liu, 2020). Typical phytoestrogens like resveratrol and quercetin have been confirmed to have high affinity for GPER, suggesting they may mediate rapid signaling through this receptor (Dong et al., 2013; Maggiolini et al., 2004).

## 6 Phytoestrogens in traditional Chinese medicine impact chronic kidney disease

In recent years, CKD has been on the rise in terms of prevalence. Its incidence and mortality rates have stayed high, placing a significant burden on patients, their families, and society at large. Various factors contribute to the development of CKD, including diabetes, hypertension, and obesity. Regardless of the underlying cause, CKD progresses gradually, leading to irreversible damage to renal cells, which can ultimately result in renal failure and, consequently, death (Ruiz-Ortega et al., 2020). Recently, angiotensin-converting enzyme inhibitors (ACEIs), angiotensin receptor blockers (ARBs), and novel renin-angiotensin-aldosterone system (RAAS) inhibitors have been the primary treatment methods for CKD. Numerous experiments and clinical studies have shown that RAAS inhibitors prevent proteinuria, renal fibrosis, and the gradual decline of renal function. Thus, they exert protective effects in both the early and late stages of kidney disease. Additionally, they reduce the incidence of major causes of death in patients with CKD, particularly cardiovascular (CV) events like congestive heart failure and myocardial infarction, as well as the mortality rate of cerebrovascular events (Alshahrani, 2023). However, with the increased use of ACEIs, ARBs, and new RAAS inhibitors, their limitations are becoming increasingly evident. For instance, some patients may experience renal function deterioration, hyperkalemia, and the phenomenon of ‘aldosterone escape’ (Zhang et al., 2017). In clinical practice, individualized treatment is crucial. Before initiating therapy, patient selection must be rigorous, and baseline indicators—such as estimated glomerular filtration rate (eGFR), serum potassium (K^+^) levels, blood pressure, and volume status—must be comprehensively assessed. During treatment, close monitoring of renal function and electrolyte changes is essential to reduce the risk of drug-related nephrotoxicity and hyperkalemia, thereby maximizing benefits and minimizing risks. Research indicates that even patients with CKD may experience AKI during monotherapy with RAAS inhibitors. Therefore, in advanced patients, careful dose adjustments are necessary to reduce proteinuria while maintaining renal perfusion (Burnier, 2020; Chou et al., 2017). With the advent of new medications, the treatment landscape for chronic kidney disease has changed significantly. Sodium–glucose cotransporter 2 inhibitors (SGLT2i), initially used for the treatment of diabetes, have now been shown to provide significant renal and cardiac protective effects, regardless of whether patients have diabetes (Podestà et al., 2023; Zebrowska and Borowiec, 2025). Additionally, nonsteroidal mineralocorticoid receptor antagonists (nsMRAs), such as finerenone, have been shown to effectively slow the progression of diabetic nephropathy and reduce the incidence of cardiovascular events (Kawanami et al., 2021; Shah et al., 2023). Glucagon-like peptide-1 receptor agonists (GLP-1 RAs) also offer renal benefits for patients with type 2 diabetes (Granata et al., 2022; Chen et al., 2025). Despite these advancements, there remains an ongoing search for complementary therapies that have multi-target effects and good safety profiles. In this context, the role of TCM containing phytoestrogen active substances in treating CKD has drawn considerable attention. Numerous experiments have demonstrated that these TCM exhibit significant therapeutic effects in managing kidney diseases (Table 1). Current experimental and clinical research reports classify the phytoestrogens derived from botanical drugs into six main categories according to their chemical structures: flavonoids, coumarins, lignans, terpenes, steroids, and stilbenes (Li J. et al., 2023) (Table 2).

**TABLE 2:** Molecular mechanisms by which phytoestrogens play a protective role in CKD.

Classification	Phytochemical	Model	Dosage	Mechanism	References
Flavonoid	Baicalin	Mouse sepsis model	200 mg/kg/days	Modulating of the BAX/BCL2 expression, inhibiting renal cell apoptosis	Zhu et al. (2016)
		Lipopolysaccharide (LPS)induce an HK-2 cell inflammatory injury model	5, 15, 25, 50,75 μmol/L	The expression of miR-223-3p was upregulated, inhibiting the TXNIP/NLRP3 signaling pathway	Sun et al. (2020a)
		TGF-β1-stimulated HK-2 cells and adriamycin (ADR)-induced FSGS model	5 μM and 50 mg/kg/d	Targeting the TGF-β1 -mediated EMT signaling pathway, downregulation of the Notch-Snail pathway	Dou et al. (2020)
		High glucose HK-2 cells and db/db mice, a model of type 2 diabetes that develops DKD	50 μM and 50 mg/kg/days	Suppressing the inflammatory responses, inhibiting of TGF-β/Smad signaling	Hu et al. (2024)
		Folic acid-induced nephropathy model and UUO mouse model	300 μM and 500 mg/kg/d	Inhibiting of TGF-β/Smad signaling, activation of CPT1A enhances fatty acid oxidation (FAO)	Miguel et al. (2023)
		db/db mouse spontaneous DN model	400 mg/kg	Activating Nrf2 signaling pathways, suppressing the oxidative stress, inhibiting the MAPK pathway	Ma et al. (2021)
	Puerarin	UUO-induced mouse model of CKD	50, 100 mg/kg/days	Modulating of the NF-κB/TGF-β1/STAT3 signaling pathway, inhibit the recruitment of inflammatory factors and the deposition of ECM	Wang et al. (2021)
		The hypoxia-reoxygenation model of HK-2 cells and rat renal ischemia-reperfusion model	1, 10 μM and 50, 100 mg/kg	Suppressing the oxidative stress and ferroptosis, inhibits the TLR4/Nox4 pathway	Jian et al. (2023)
		The pyroptosis model of podocytes induced by high glucose and Streptozotocin (STZ)-induced DN rats	0.8 mM and 80 mg/kg	Upregulated SIRT1 and inhibited TXNIP/NLRP3 inflammasome activation, Inhibits the Caspase-1 pathway	Wang et al. (2025)
Coumarin	Psoralen	UUO mouse model	20 mg/kg	Targeting the TGF-β1/Smad2/3 EMT signaling pathway, Inhibit the NLRP3 inflammasome pathway	Lee et al. (2023)
		Meso-13 mesangial cells were treated with high glucose and STZ induces diabetic mice	4, 50, 200 μg/mL and 500 mg/kg/days	Inhibiting the TGF-β signaling pathway, Inhibit caspase activation/PARP cleavage	Seo et al. (2017)
	Osthole	High glucose induces HBZY-1 mesangial cells and T2DM rats (STZ/high fat and high sucrose)	1, 5, 10 μM and 25, 50, 100 mg/kg	Inhibiting the TGF-β/Smads/NF-κB signaling pathway	Li et al. (2024a)
		HK-2 cells induced by TGF-β1 and Interleukin-11 (IL-11) and UUO mouse model	100 μM and 10, 20 mg/kg	Inhibiting the TGF-β/Smad2/3 signaling pathway, targeting the IL-11/ERK1/2 pathway improves the kidneys	Wu et al. (2021)
		Advanced Glycation End products (AGEs) induce HK-2 cells	5, 20, 100 μM	Inhibition of the JAK2-STAT1/3 signaling pathway mediated by AGEs/RAGE	Kan et al. (2019)
Lignan	Schisandra chinensis stem extract (SCE)	Cisplatin-induced AKI model in ICR mice	300,600 mg/kg	Inhibiting the NF-κB/caspase signaling pathway	Li et al. (2018)
	Schisandrin A	HK-2/NIH-3T3 cells induced by TGF-β and UUO mouse model	10–40 μM and 20, 40 mg/kg/days	Suppressing the oxidative stress, downregulated PKCβ expression	Liu J. et al. (2024)
	Schisandrin B	Gentamicin-induced renal toxicity rat model	1–10 mg/kg/days	Enhance the antioxidant capacity of mitochondria, improve mitochondrial function/structural integrity	Chiu et al. (2008)
		NRK-52E cells and Wistar rats	6.25 μM and 10 mg/kg/days	Suppressing the oxidative stress, regulate the renin-angiotensin system	Stacchiotti et al. (2011)
Stilbene	Resveratrol	High-fat diet (HFD)-induced hyperuricemia (HUA) and kidney injury model	100 mg/kg/days	Promoting the proliferation of beneficial intestinal flora that degrade UA, improving purine metabolism-related pathways, inhibiting harmful proinflammatory bacteria	Zhou et al. (2024)
		Primary rat mesangial cells	10 μM	Suppressing the oxidative stress, protect mitochondrial function	Xu et al. (2012)
		Rat mesangial cell line and Primary rat mesangial cells	0.1–10 μM	Inhibiting the NF-κB signaling pathway	Zhang et al. (2012)
		Rat mesangial cells and Streptozotocin (STZ) induced type 1 diabetic mouse model	25 μM and 10 mg/kg/days	Inhibiting the Akt/NF-κB signaling pathway	Xu et al. (2014)
	2,3,5,4′-tetrahydroxystilbene-2-O-β-d glucoside (THSG)	MES13 mesangial cells and Adriamycin (ADR)-induced FSGS model	0.4–1.6 μg/mL and 2.5, 10 mg/kg	Activates the Nrf2-Keap1 pathway, suppressing the oxidative stress	Lin et al. (2018)
		Streptozotocin (STZ)-induced diabetic mouse model	10,40 mg/kg	Inhibiting the TGF-β signaling pathway, activate the protective ANG (1–7)/Mas axis	Chen X. et al. (2016)
Terpenoid	Ginsenoside Rh1 (G-Rh1)	High-fat diet (HFD)/Streptozotocin (STZ) induced DN mouse	5, 10 mg/kg	Modulating of the AMPK/PI3K/Akt signaling pathway	Su et al. (2021)
	Ginsenoside Rb1 (Rb1)	CKD model induced by adenine	40 mg/kg/days	Activates the PPAR-γ pathway, inhibiting the Wnt/β-catenin signaling pathway	Zhou et al. (2019)
Sterol	Dioscin	NRK-52E cells and SD rats, BALB/c mice	25–200 nmol/L and 15, 30, 60 mg/kg, 80 mg/kg	Activates the Nrf2 pathway, suppressing the oxidative stress, inflammatory responses	Li et al. (2021a)
		High-fat diet (HFD) and Streptozotocin (STZ) induced type 2 diabetic rats	20 mg/kg	Inhibiting oxidative stress, inflammation, and apoptosis mediated by the mitochondria and ER stress, modulating of the AMPK/mTOR signaling pathway	Zhong et al. (2022)
		Cisplatin-induced AKI rats	60 mg/kg	Modulating of the Nrf2/HO-1/NF-κB signaling pathway, inhibiting oxidative stress, inflammation	Wang et al. (2024)
		MPC5 podocyte cell line and db/db DN mice	0.1, 1 μM and 30,90 mg/kg/days	Regulate SIRT6, reduce lipid accumulation, and protect podocytes	Wang et al. (2022)

### 6.1 Flavonoids

Flavonoids are natural small-molecule products consisting of two benzene rings (A and B) connected by a heterocyclic pyranone (C). They are present in a wide variety of plants (Sun et al., 2022). Flavonoid PE, one of the most common and extensively studied phytoestrogens, mainly comprises flavones and isoflavones, and serves as significant raw materials in the fields of nutrition, medicine, and cosmetics. Research demonstrates that baicalin can exert a renal protective effect through multiple mechanisms. With respect to apoptosis, baicalin can inhibit this process and mitigate kidney damage by down-regulating the expression of the pro-apoptotic protein BCL2-associated X protein (BAX) and up-regulating the expression of the anti-apoptotic protein b-cell cell lymphoma 2(BCL2) (Zhu et al., 2016). In terms of inhibiting inflammatory responses, baicalin can also enhance the expression of microRNA-223-3p (miR-223-3p) and suppress the activation of the thioredoxin-interacting protein (TXNIP)/nucleotide-binding domain, leucine-rich repeat containing pyrin domain containing 3(NLRP3) inflammatory signaling pathway, thus diminishing inflammatory reactions (Sun et al., 2020a). Moreover, baicalin can inhibit the EMT of podocytes by repressing the Notch1-Snail axis, markedly reducing adriamycin-induced glomerular damage in mice and lowering proteinuria levels (Dou et al., 2020). Additionally, studies have revealed that baicalin can inhibit the expression of inflammatory and fibrotic genes induced by TGF-β, while concurrently boosting fatty acid oxidation (FAO) levels by activating carnitine palmitoyl transferase 1A (CPT1A), thereby enhancing kidney energy metabolism and effectively alleviating renal fibrosis (Hu et al., 2024; Miguel et al., 2023). Diabetic nephropathy (DN) is one of the primary microvascular complications of diabetes and has emerged as the leading cause of CKD in China. Oxidative stress and inflammation are crucial factors in the onset and progression of DN. Research indicates that baicalin can significantly lower blood glucose levels in db/db mice and decrease urinary albumin excretion. This effect may be associated with the activation of the nuclear factor erythroid 2-related factor 2 (Nrf2)-mediated antioxidant signaling pathway and the inhibition of the mitogen-activated protein kinase (MAPK)-mediated inflammatory signaling pathway (Ma et al., 2021).

Puerarin is a hydroxyisoflavone with the molecular formula C21H20O9. This metabolite is found in various plants and botanical drugs, including *Pueraria montana* var. *lobata* (Willd.) Sanjappa & Pradeep (Fabaceae). It has been extensively studied for its estrogenic effects. Research indicates that puerarin exhibits significant kidney-protective properties. A study conducted by Wang et al., in 2021 demonstrated that in a renal fibrosis model induced by UUO, puerarin effectively inhibits the expression of inflammatory factors interleukin-1β (IL-1β), interleukin-6 (IL-6), and monocyte chemoattractant protein-1 (MCP-1), reduces ECM deposition, alleviates inflammatory and fibrotic reactions, and ultimately improves renal function by regulating the NF-κB p65/STAT3 and TGF-β1/Smads signaling pathways (Wang et al., 2021). Furthermore, in experiments involving intraperitoneal injection of puerarin (50 or 100 mg/kg) prior to renal ischemia-reperfusion in rats, it was observed that puerarin pretreatment reduced the expression of the renal fibrosis marker α-smooth muscle actin (α-SMA) in a dose-dependent manner. When HK-2 cells were subjected to hypoxia/reoxygenation, the expression of α-SMA significantly increased. Similarly, puerarin pretreatment (1 µM or 10 µM) also attenuated this increase in a dose-dependent manner. These findings indicate that puerarin possesses the ability to alleviate renal fibrosis in both *in vivo* and *in vitro* models. Puerarin has demonstrated significant antioxidant capacity in animal experiments (Jian et al., 2023). It upregulates the levels of antioxidant enzymes such as superoxide dismutase (SOD), glutathione, and catalase, while simultaneously reducing the levels of malondialdehyde. This action effectively mitigates oxidative stress-induced damage to the kidneys. Dyslipidemia is a common complication of chronic kidney disease and is closely associated with the deterioration of renal function. In animal model experiments, puerarin has been shown to regulate blood lipid levels, significantly reducing triglycerides, total cholesterol, and low-density lipoprotein cholesterol, thereby delaying the progression of chronic kidney disease (Xu et al., 2025). Podocyte injury is a primary pathological process in diabetic nephropathy. Studies indicate that puerarin may inhibit podocyte pyroptosis, reduce podocyte injury, and alleviate renal inflammatory damage by regulating the silent mating type information regulation 2 homolog 1 (SIRT1)/nucleotide-binding domain, leucine-rich repeat containing pyrin domain 3 (NLRP3)/cysteine-aspartic acid protease 1 (Caspase-1) pathway (Wang et al., 2025).

### 6.2 Coumarins

Coumarin metabolites, characterized by their aromatic odors, are a class of natural products widely distributed throughout the plant kingdom, found in families including Umbelliferae, Rutaceae, Asteraceae, Leguminosae, and Orchidaceae (Lončar et al., 2020; Tang et al., 2024). In recent years, traditional Chinese medicines and proprietary Chinese medicines containing coumarin metabolites have been increasingly utilized in clinical treatments. Coumarin PE, an aromatic chemical with a benzopyranone structure, has angelicin and psoralen as typical representatives of this metabolite class. Despite their structural similarities, their mechanisms of action differ significantly. Angelicin has been shown to activate the NF-κB pathway, which contributes to its anti-inflammatory effects in various diseases (Mahendra et al., 2020). The NF-κB pathway is also crucial in the context of chronic kidney diseases, positioning angelicin as a potential phytoestrogen in TCM. However, research on its effects on kidney diseases remains limited, necessitating future investigations to enhance our understanding of its role in kidney health. In contrast, psoralen exerts anti-inflammatory effects by inhibiting the production of nitric oxide (NO) (Y. J. Kim et al., 2016).

In traditional medicine, psoralen has been widely used for treating a range of conditions, including inflammatory and fibrosis-related diseases (Ren et al., 2020). The inflammatory response is a critical pathological mechanism in the progression of chronic kidney disease, which can activate the nucleotide-binding oligomerization domain, leucine-rich repeat and pyrin domain-containing protein 3(NLRP3) inflammasome, leading to renal tissue damage and fibrosis. Research findings indicate that psoralen can not only reduce the activation of the NLRP3 inflammasome in UUO mice and decrease the expression of downstream cytokines, but it can also inhibit the TGF-β1/Smad pathway. This inhibition subsequently leads to a reduction in the expression level of the fibrotic marker α-smooth muscle actin (α-SMA) and alleviates renal fibrosis induced by UUO (Lee et al., 2023). Psoralen is the primary metabolite of the seed extract from *Cullen corylifolium* (L.) Medik. (formerly *Psoralea corylifolia* L.). After administering psoralen extract (500 mg/kg/day) orally for 8 weeks to streptozotocin -induced diabetic mice, notable decreases were observed in creatinine clearance, urine volume, urinary microalbumin, and mesangial expansion, alongside a significant reduction in renal tissue fibrosis in diabetic mice. *In vitro* experiments demonstrated that both the psoraleae corylifoliae semen (PCS) extract and its main metabolite, psoralen, significantly enhanced the viability of high-glucose-treated glomerular mesangial cells and reduced the expression of apoptosis-related proteins and fibrosis-related genes [such as TGF-β1, FN, and plasminogen activator inhibitor-1(PAI-1)]. Moreover, the expression of anti-apoptotic proteins (including Bcl-2 and phosphorylated Bad) was also increased (Seo et al., 2017). These studies suggest that, following appropriate clinical trials, this traditional Chinese botanical drug phytoestrogen has the potential for widespread application in the treatment of chronic kidney disease.

*Cnidium monnieri* (L.) Cusson (Apiaceae), known in Chinese as She Chuang Zi, is an important botanical drug that has been used in China for centuries to treat chronic kidney disease, female genital issues, male impotence, and frigidity (Sun et al., 2020b). Its biological activity is mainly attributed to osthole, a coumarin-based TCM metabolite. Researchers established a type 2 diabetes rat model induced by streptozotocin combined with a high-fat and high-sugar diet, using metformin as a positive control, to explore the therapeutic effects of osthole on diabetic nephropathy. After 8 weeks of intervention treatment, the study found that *Cnidium monnieri* can reduce the increase of ROS in high glucose-induced glomerular mesangial cells and downregulate the expression of the TGF-β1/Smads signaling pathway and related proteins, thus exerting a preventive and therapeutic effect on diabetic nephropathy (Li et al., 2024c). In research on a mouse renal fibrosis model, osthole demonstrated significant anti-fibrosis effects through multiple mechanisms. On one hand, it effectively inhibits the renal fibrosis process by blocking the TGF-β/Smad signaling pathway; on the other hand, osthole can also directly act on the interleukin-11 (IL-11)/extracellular signal–regulated kinase 1/2 (ERK1/2) signaling pathway to inhibit the translation of fibrotic proteins, thereby improving renal fibrosis (Wu et al., 2021). *In vitro* experiments have further confirmed the effects of osthole on rat renal interstitial fibroblasts normal rat kidney fibroblast cell line (NRK-49F). Osthole can inhibit the activation of NRK-49F cells and significantly reduce the expression of α-SMA, FN, and collagen I, thereby decreasing the production of extracellular matrix. Additionally, osthole can inhibit the proliferation of NRK-49F cells, contributing to the improvement of renal fibrosis from multiple aspects (Zhang et al., 2018). Research has identified that a significant characteristic of diabetic nephropathy is the massive accumulation of advanced glycation end products (AGEs) in renal tissue. AGEs bind to receptors receptor for advanced glycation end products (RAGEs), activating multiple intracellular signaling pathways that trigger oxidative stress responses, leading to tubular interstitial hypertrophy and fibrosis. Furthermore, studies have shown that osthole can inhibit the activation of the AGE/RAGE-induced janus kinase 2 (JAK2)- signal transducer and activator of transcription 1/3(STAT1/3) signaling pathway by inducing the expression of Klotho protein, reducing the expression of p21Waf1/Cip1, collagen IV, and RAGE protein, effectively inhibiting AGE-induced tubular hypertrophy and protecting the kidneys (Kan et al., 2019). The expression level of Klotho is closely related to the progression of CKD (Liu J. et al., 2024). Osthole may treat chronic kidney disease by regulating Klotho expression, although its mechanism of action requires further investigation.

### 6.3 Lignans

Lignan phytoestrogens are widely present in various diets, including cereal bran, beans, flaxseed, sesame, and unrefined grains (Rizzolo-Brime et al., 2022). Schisandrin A (SchA) and Schisandrin B(SchB), both essential active metabolites of *S. chinensis* (Turcz.) Baill. (Schisandraceae), belong to lignan metabolites. Data indicate that *Schisandra chinensis* is considered a natural dietary supplement for protecting kidney function. In an experimental model of cisplatin-induced acute kidney injury in mice, the Schisandra chinensis stem extract (SCE), primarily composed of lignan metabolites, demonstrated significant multi-target renal protection. Firstly, SCE effectively improved renal function indicators by significantly reducing serum creatinine and blood urea nitrogen levels. Regarding apoptotic regulation, SCE significantly inhibited the expression of the pro-apoptotic protein Bax while simultaneously upregulating the expression of the anti-apoptotic protein Bcl-2, thereby bidirectionally regulating the expression of apoptotic-related proteins and effectively reducing the apoptosis of renal tubular epithelial cells. In terms of antioxidant and anti-inflammatory effects, SCE reduced the generation of lipid peroxidation products (such as Malondialdehyde) and increased the content of antioxidants (such as Glutathione) to alleviate oxidative damage to renal cells. Additionally, it inhibited the expression of inflammatory mediators’ inducible nitric oxide synthase (iNOS) and cyclooxygenase-2 (COX-2) and the activation of the NF-κB signaling pathway, thereby reducing kidney damage caused by inflammatory reactions (Li et al., 2018). Molecular docking and cellular thermal shift analysis show that SchA directly binds to the PKCβ protein and inhibits its activity. This inhibition subsequently reduces the levels of fibrotic markers, such as FN, collagen I, vimentin, and α-smooth muscle actin. Additionally, SchA inhibits the proliferation and differentiation of fibroblasts, thereby mitigating the progression of renal fibrosis (Liu H. L. et al., 2024). SchB has also been shown to ameliorate renal damage induced by mercury and arsenic (Chiu et al., 2008; Stacchiotti et al., 2011). The lignan active metabolites, SchA and SchB, found in *S. chinensis*, exhibit significant protective potential against CKD through the synergistic action of multiple targets and pathways.

### 6.4 Stilbene

Stilbene, a polyphenolic organic metabolite of plant origin with the chemical formula C_14_H_12_ (Dubrovina and Kiselev, 2017), has a carbon skeleton of 1,2-diphenylethylene (C6–C2–C6), formed by connecting two benzene rings *via* an ethylene bridge. Grapes, peanuts, berries, and certain botanical drugs are the main sources of stilbene (Al-Khayri et al., 2023). Among stilbene metabolites, resveratrol has been the subject of extensive research, particularly for its anti-inflammatory properties. Yu Qinzhou (Zhou et al., 2024) and colleagues found that resveratrol can improve glomerular atrophy and tubular structure, reduce renal fibrosis and inflammation, and ultimately alleviate hyperuricemia and associated renal injury. This is achieved by inhibiting liver xanthine oxidase activity and decreasing the expression of renal inflammatory factors such as IL-6 and TNF-α. Resveratrol is abundant in *Reynoutria japonica* Houtt. (Polygonaceae), a commonly used nephrology medication for treating acute and chronic renal failure. Meta-analyses have provided definitive evidence of resveratrol’s renal protective effect in adults (Abdollahi et al., 2023). The protective mechanism primarily involves activating the SIRT1 (silent information regulator 1) pathway, enhancing mitochondrial function, and reducing ROS production (Guarente, 2011; Kitada et al., 2013). Additionally, it effectively inhibits the mechanistic target of rapamycin (mTOR) pathway associated with renal injury in mammals (Inoki et al., 2011; Liu et al., 2010; Sakaguchi et al., 2006). In addition, resveratrol can protect renal mitochondria from glucose-induced oxidative stress damage by maintaining mitochondrial complex III activity (Xu et al., 2012), inhibiting c-Jun N-terminal kinase (JNK) and NF-κB activation (Zhang et al., 2012), and down-regulating plasminogen activator inhibitor-1 expression (Xu et al., 2014). Although cell and animal experiments have shown that resveratrol has various potential benefits, its effects in humans need further research verification, and it should be used with caution in practical applications.

End-stage renal disease represents a more advanced and challenging stage of chronic kidney disease. The primary pathological features include progressive glomerular sclerosis and renal interstitial fibrosis (Xie et al., 2023). The main active metabolite in *R. multiflora* (Thunb.) Moldenke (Polygonaceae), 2,3,5,4′-tetrahydroxystilbene-2-O-β-D-glucoside (TSG), exhibits significant renal protective effects. In the study investigating the mechanism of glomerular sclerosis improvement, mice were continuously treated with oral gavage of TSG at doses of 2.5 and 10 mg/kg for 24 days, while a single intravenous dose of adriamycin (AD, 10 mg/kg) was administered on the third day. The experimental results indicate that TSG can maintain the expression level of podocin, a podocyte marker, mitigate AD-induced podocyte damage, and ultimately reduce the occurrence of proteinuria and the formation of glomerular sclerosis. In terms of renal fibrosis improvement, TSG significantly diminishes oxidative stress levels by activating the nuclear factor erythroid 2-related factor 2- Kelch-like ECH-associated protein 1(Nrf2-Keap1) antioxidant pathway. This mechanism of action results in decreased mRNA and protein expression levels of fibrosis markers in the kidneys, thereby effectively alleviating the renal fibrosis process induced by AD and providing renal protection in the AD-induced Focal segmental glomerulosclerosis mouse model (Lin et al., 2018). In many regions, diabetes is the leading cause of ESRD (Reutens and Atkins, 2011). Between 25% and 50% of diabetic patients may develop CKD, commonly referred to as DN (Li et al., 2016; Plantinga et al., 2010). In Streptozotocin induced diabetic models, TSG can inhibit the expression of downstream profibrotic and proinflammatory factors (such as TGF-β, CTGF, MCP-1, *etc.*) by blocking the activation of the RAS and reducing the accumulation of angiotensin II. Furthermore, TSG can restore the expression of key structural proteins of the glomerular filtration barrier, thereby reducing proteinuria and tubulointerstitial fibrosis (Chen G. T. et al., 2016). As a botanical drug with various clinical pharmacological benefits, *Reynoutria multiflora* holds potential for preventing the progression of CKD and has significant clinical application value.

### 6.5 Terpenoids

Terpenoids are a class of organic metabolites that are widely present in nature. They are composed of isoprene units and exhibit diverse biological activities (Chen et al., 2011). Among the extensive family of terpenoids, certain members with specific structures have been identified to possess estrogenic activity and regulate estrogen receptors (Hsu et al., 2011). *Panax ginseng* C.A. Mey. (Araliaceae), a staple in Eastern medicine, is notable for its high content of triterpene saponins and other active metabolites, which confer pharmacological effects such as enhanced immunity and prevention of chronic diseases (Osbourn et al., 2011; Ratan et al., 2021). Given the substantial evidence supporting its efficacy, *P. ginseng* and its extracts have transitioned from traditional Eastern remedies to natural medicines that are increasingly acknowledged within the Western medical system (Li Z. et al., 2023).

The primary active metabolites in *P. ginseng*, including ginsenoside Re, Rg1, Rg3, Rh1, and Rb1, all belong to the triterpenoid class and exhibit estrogen-like activity (Li et al., 2024a). In the context of treating kidney diseases, G-Rh1 has been shown to significantly reduce the expression of Bax and cleaved caspase 3 and caspase 9 in the renal tissue of DN mice. Concurrently, it upregulates the expression of Bcl-2 and Bcl-XL, indicating its anti-apoptotic effects. Histological analysis *via* H&E staining revealed a reduction in pathological damage to the renal tissue of DN mice, including thickening of the glomerular basement membrane and glomerular atrophy. These findings collectively suggest the protective effect of G-Rh1 on the kidneys (Su et al., 2021). Furthermore, in terms of antioxidant and anti-inflammatory effects, ginsenosides can significantly enhance the expression levels of antioxidant enzymes by activating the Nrf2/ARE signaling pathway, thereby effectively mitigating oxidative damage. They also alleviate renal inflammatory responses by inhibiting the activation of the NF-κB signaling pathway and reducing the expression of inflammatory factors. More importantly, ginsenosides can also regulate the TGF-β1/Smad signaling pathway, inhibit excessive deposition of extracellular matrix, and block the progression of renal fibrosis. Thus, they play a protective role in glomerular filtration function and tubular reabsorption function (Fan et al., 2023). Vascular calcification (VC), a strong prognostic marker for cardiovascular disease mortality, is commonly observed in CKD. Studies have shown that CKD patients are prone to VC even in the early stages, with a prevalence of 25% in stage 3 and 35% in stage 4. Once CKD patients begin dialysis, the prevalence of VC rises rapidly, exceeding 50% (Russo et al., 2004; Sigrist et al., 2007). G-Rb1 can not only alleviate the progression of early CKD by regulating oxidative stress and inflammation (Xu et al., 2017), but it can also improve CKD-related VC by activating peroxisome proliferator-activated receptor-γ (PPAR-γ) to inhibit the Wnt/β-catenin pathway (Zhou et al., 2019). These comprehensive effects demonstrate that ginseng exhibits significant protective effects in various kidney disease models.

### 6.6 Sterol

**FIGURE 1:**
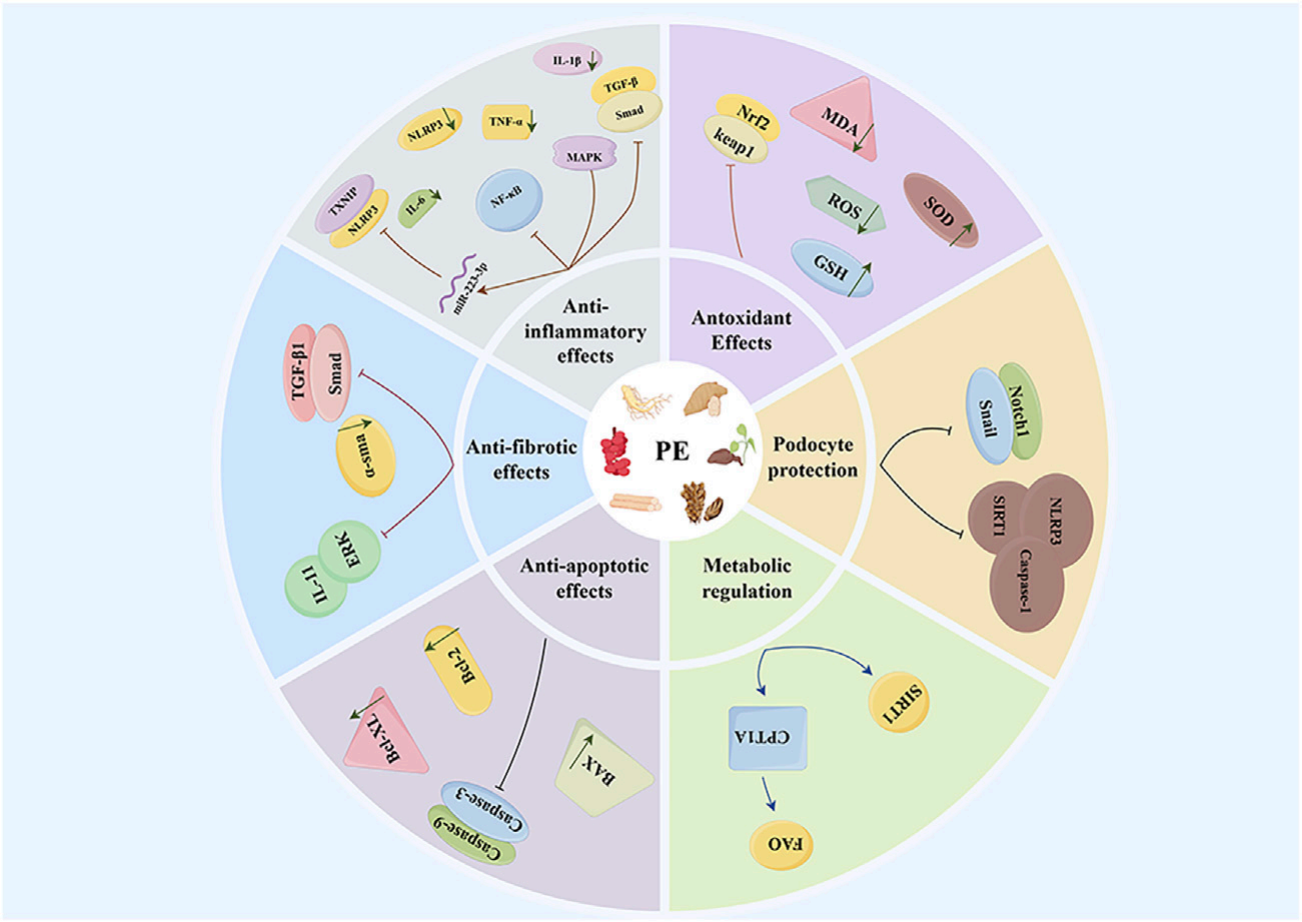
The mechanisms by which phytoestrogens from traditional Chinese medicine affect CKD.

Dioscin, a natural steroidal saponin metabolite primarily found in *Dioscorea panthaica* Prain & Burkill (Dioscoreaceae), belongs to a class of phytoestrogens that perform various functions, including endocrine regulation and anti-inflammatory effects (Tang et al., 2015; Yang et al., 2019). Recent research has demonstrated that dioscin possesses significant antioxidant activity and lipid-lowering effects (Mao et al., 2023). In the context of CKD, although research is relatively limited, experimental evidence suggests that dioscin can function as a phytoestrogen and exert renal protective effects through multiple mechanisms. Notably, dioscin reduces the expression level of microRNA-145-5p (miR-145-5p), thereby inhibiting miR-145-5p-mediated oxidative damage. Concurrently, it decreases the levels of the oxidative stress product malondialdehyde (MDA) while increasing the levels of glutathione (reduced form) (GSH) and glutathione peroxidase (GSH-Px), which collectively improve methionine-induced liver and kidney injury (Li et al., 2021a). Furthermore, it has been reported that dioscin can inhibit renal cell apoptosis by enhancing the quality and quantity of mitochondria, thus reducing renal injury in diabetic nephropathy models (Zhong et al., 2022). In chronic kidney disease, both apoptosis and necrosis of renal cells are critical factors contributing to the decline of renal function. Animal studies further confirm that dioscin treatment can significantly enhance renal function indicators, such as blood creatinine and urea nitrogen levels (Wang et al., 2024). Proteinuria is a significant marker of chronic kidney disease, and its production is closely associated with damage to the glomerular filtration barrier. Massive proteinuria can further impair glomerular filtration function, creating a vicious cycle between the two (Makhammajanov et al., 2024). Podocytes, which are highly differentiated epithelial cells, surround the glomerular capillaries. Alterations in their foot process structure, such as fusion or disappearance, are critical factors leading to proteinuria. Therefore, safeguarding podocyte function is crucial for preventing and mitigating proteinuria (Nagata, 2016). Dioscin has been shown to protect podocytes from damage and reduce proteinuria by regulating SIRT6 and diminishing lipid accumulation (Wang et al., 2022). These findings provide a scientific basis for considering dioscin as a potential therapeutic agent for chronic kidney disease (Figure 1).

## 7 Perspectives and conclusion

Natural phytoestrogens present in TCM resemble endogenous estrogens structurally and can bind to estrogen receptors, thereby exerting estrogen-like effects. They exhibit multiple pharmacological effects—anti-inflammatory, antioxidant, anti-fibrotic, and immunomodulatory—showing promise for CKD treatment. Research shows that various phytoestrogens from TCM can benefit chronic kidney disease through multiple mechanisms. Specifically, these mechanisms involve inhibiting pro-inflammatory factors (e.g., IL-6, and NF-κB), suppressing oxidative stress responses, blocking inflammatory signaling pathways, reducing cell apoptosis, improving renal fibrosis, and decreasing proteinuria. These multi-target effects provide a theoretical basis for the clinical use of phytoestrogens. However, applying these laboratory findings in clinical settings remains challenging. The transition from basic research to clinical applications continues to face obstacles. Current studies primarily focus on non-estrogen receptor-related mechanisms, while the interaction mechanisms between phytoestrogens and estrogen receptors remain underexplored. Future research should systematically clarify their target pathways and comprehensively evaluate their safety and efficacy.

Notably, in 1999, the U.S. Food and Drug Administration (FDA) approved phytoestrogens, specifically soy isoflavones from soybeans, for use (Lee, 2006). A randomized controlled trial (RCT) evaluated a phytoestrogen-containing metabolite, soy isoflavones, for treating menstrual migraines. It found this combination significantly outperformed placebos in preventing menstrual-related migraines (Burke et al., 2002). Recently, TCM has gained global healthcare prominence. Yet, safety concerns, especially nephrotoxicity risks, persist due to its complex composition, influenced by botanical drug type, dosage, usage duration, and individual health (Yang et al., 2018). For example, TCM with aristolochic acid links to renal failure and urinary tract tumors. Similarly, *Cassia obtusifolia* L. (Fabaceae), while aiding constipation and eye issues, can cause renal damage when overused (Komatsu et al., 2025; Yang et al., 2024). Thus, phytoestrogens clinical use must ensure efficacy, clarify safe dosage thresholds, and strengthen toxicological research.

Compared to Chinese herbal compound, phytoestrogens with clear sources and single components carry a lower risk of toxic interference and can somewhat avoid potential nephrotoxicity. However, their use should be evaluated from multiple perspectives, with dosage controlled to ensure safety and efficacy within an appropriate range. Additionally, suitable usage guidelines should be established for individuals with existing kidney diseases to ensure efficacy while minimizing adverse reactions, thereby providing safer and more effective treatment options for CKD patients. Although phytoestrogens show great potential in treating chronic kidney disease, current research faces several challenges. Most studies on the renal protective effects of plant estrogens in TCM are limited to *in vitro* experiments and animal models, lacking large-scale, randomized controlled trials to verify their safety and efficacy in clinical settings. Existing clinical trials have small sample sizes and short follow-up periods, making it difficult to draw clear conclusions about the long-term effects and potential side effects of phytoestrogens in CKD patients. Furthermore, due to genetic differences, metabolic characteristics, and variations in baseline health conditions, individual responses to plant estrogens exhibit significant variability, which current research has not yet adequately considered. Although short-term use shows good tolerance, the safety of long-term use of plant estrogens in CKD patients and their potential estrogen-like effects on other organ systems have not been fully assessed. While the mechanisms of action of plant estrogens are beginning to be understood, there are still significant gaps in knowledge regarding how these metabolites interact with kidney cell types and signaling pathways.

To promote further development in this field, future research should prioritize several key directions. First, conducting large-scale randomized controlled trials will be of vital importance to evaluate the efficacy and safety of phytoestrogens in patients with CKD. These trials should cover diverse patient populations and feature longer follow-up periods to better assess long-term outcomes. Additionally, it is crucial to delve into the genetic and metabolic factors that influence individual responses to phytoestrogens. This exploration paves the way for developing personalized treatment strategies tailored to these factors. Moreover, long-term safety studies must be executed to uncover and mitigate any potential side effects of phytoestrogen use in CKD patients, while also gauging the overall safety of extended usage. Simultaneously, in-depth mechanistic research is imperative to enhance our comprehensive understanding of the interplay between phytoestrogens and renal cells and signaling pathways. Finally, research should also be directed toward exploring how phytoestrogens can beneficially combine with current CKD treatment methods. This includes looking into synergistic effects and how such combinations might lead to improvements in therapeutic outcomes.

In summary, phytoestrogens, as natural bioactive metabolites derived from TCM, hold great research value in preventing and treating chronic kidney disease. They have the potential to offer CKD patients a safer and more effective novel therapeutic strategy.

## Glossary

TCM: traditional Chinese medicine
PE: phytoestrogens
CKD: chronic kidney disease
ESRD: end-stage renal disease
RAS: renin-angiotensin system
GPER: G protein-coupled estrogen receptor
Erα: Estrogen receptor α
ERβ: Estrogen receptor β
PI3K: phosphatidylinositol 3-kinase
AKI: acute kidney injury
CRP: C-reactive protein
ER: estrogen receptor
ECM: extracellular matrix
TGF-β: transforming growth factor-β
FN: fibronectin
ROS: reactive oxygen species
NF-κB: nuclear factor-κB
MAPK: mitogen-activated protein kinase
IL-6: interleukin-6
TNF-α: tumor necrosis factor-α
eGFR: estimated glomerular filtration rate
FSH: follicle-stimulating hormone
PAI-1: plasminogen activator inhibitor-1
HK-2 cells: human proximal tubular epithelial (HK-2) cells
SMAD: Sma- and Mad-related protein
ACEIs: angiotensin-converting enzyme inhibitors
ARBs: angiotensin receptor blockers
CV: cardiovascular
BAX: BCL-2-associated X protein
BCL-2: B-cell lymphoma-2
miR-223-3p: microRNA-223-3p
TXNIP: thioredoxin-interacting protein
EMT: epithelial-mesenchymal transition
FAO: fatty acid oxidation
CPT1A: carnitine palmitoyl transferase 1A
DN: diabetic nephropathy
UUO: unilateral ureteral obstruction
IL-1β: interleukin-1β
MCP-1: monocyte chemoattractant protein-1
α-SMA: α-smooth muscle actin
STAT3: signal transducer and activator of transcription3
SOD: superoxide dismutase
NO: nitric oxide
NLRP3: nucleotide-binding domain, leucine-rich repeat containing pyrin domain 3
Caspase-1: cysteine-aspartic acid protease 1
IL-11: interleukin-11
ERK: extracellular signal–regulated kinase
NRK-49F: normal rat kidney fibroblast cell line
AGE: advanced glycation end products
RAGE: receptor for advanced glycation endproducts
SchA: Schisandrin A
SchB: Schisandrin B
SCE: Schisandra chinensis stem extract
MDA: malondialdehyde
GSH: glutathione
SIRT6: silent information regulator 6
iNOS: Inducible nitric oxide synthase
COX-2: cyclooxygenase-2
PKCβ: Protein Kinase Cβ
SIRT1: silent information regulator 1
mTOR: mechanistic target of rapamycin
TSG: 2,3,5,4′-tetrahydroxystilbene-2-O-β-D-glucoside
AD: Adriamycin (doxorubicin)
CTGF: Connective tissue growth factor
ZO-1: zonula occludens-1
FAT1: FAT atypical cadherin 1
GRh1: Ginsenoside Rh1
VC: vascular calcification
PPARγ: Peroxisome Proliferator-Activated Receptor γ
GSH-Px: glutathione peroxidase
HBV: hepatitis B virus
MCF-7: Michigan Cancer Foundation-7
SREBP-1c: sterol regulatory element-binding protein 1c
FASN: fatty acid synthase
ATGL: adipose triglyceride lipase
FDA: Food and Drug Administration
RCT: randomized controlled trial
SGLT2i: Sodium–glucose cotransporter 2 inhibitors
nsMRAs: Nonsteroidal mineralocorticoid receptor antagonists
GLP-1 RAs: Glucagon-like peptide-1 receptor agonists
